# Flower-Shaped Domains and Wrinkles in Trilayer Epitaxial Graphene on Silicon Carbide

**DOI:** 10.1038/srep04066

**Published:** 2014-02-11

**Authors:** B. Lalmi, J. C. Girard, E. Pallecchi, M. Silly, C. David, S. Latil, F. Sirotti, A. Ouerghi

**Affiliations:** 1 CNRS- Laboratoire de Photonique et de Nanostructures (LPN), Route de Nozay, 91460 Marcoussis, France; 2 Synchrotron-SOLEIL, Saint-Aubin, BP48, F91192 Gif sur Yvette Cedex, France; 3 CEA – Saclay, DSM/IRAMIS/SPCSI, 91191 Gif sur Yvette France

## Abstract

Trilayer graphene is of particular interest to the 2D materials community because of its unique tunable electronic structure. However, to date, there is a lack of fundamental understanding of the properties of epitaxial trilayer graphene on silicon carbide. Here, following successful synthesis of large-area uniform trilayer graphene, atomic force microscopy (AFM) showed that the trilayer graphene on 6H-SiC(0001) was uniform over a large scale. Additionally, distinct defects, identified as flower-shaped domains and isolated wrinkle structures, were observed randomly on the surface using scanning tunneling microscopy and spectroscopy (STM/STS). These carbon nanostructures formed during growth, has different structural and electronic properties when compared with the adjacent flat regions of the graphene. Finally, using low temperature STM/STS at 4K, we found that the isolated wrinkles showed an irreversible rotational motion between two 60° configurations at different densities of states.

The peculiar band structure of massive Dirac fermions in multilayer graphene is driving intense activity in fundamental research, as well as for applications in next generation optoelectronic devices. Most of these studies have been focused on ABA and ABC trilayer graphene, with their tunable electronic properties, which depend on the type of stacking^1^,^2^,^3^,^4^,^5^. For instance, exciting properties have been predicted for ABC multilayers, such as high Tc superconductivity or magnetism^6^,^7^,^8^. Nevertheless, the production of large-area trilayer graphene with a controlled structure and its characterization, which includes stacking defects, lattice distortion, and corrugations, are still largely unexplored. Several routes are currently being pursued in the synthesis of wafer- thin, uniform graphene layers. Among these, the epitaxial approach based on SiC graphitization is a viable method for large-scale graphene production^9^,^10^,^11^. A considerable advantage of this technique is that the insulating SiC wafers can be employed as a substrate so that a graphene transfer step is not required. This method has been developed on C-terminated SiC and used to grow graphene multilayers with rotational disorder and a large number of layers, typically N ~ 10–100^12^,^13^. Growth on the Si face of SiC has been developed and used to produce high quality wafer- thin monolayer graphene^10^,^11^,^14^. However, only a few papers discuss the possibility of growing a few layers of epitaxial graphene (N ~ 3–5) on the Si-terminated face^14^.

Epitaxial graphene, as well as the other large scale graphenes, can possess a variety of structural defects that can affect the material's properties. For example, a high number of substrate steps^15^,^16^ can degrade the electronic properties of epitaxial graphene films on SiC. Corrugations of the epitaxial graphene film have also been reported. These corrugations were first observed on epitaxial graphene grown on the C face of a SiC substrate, and include ripples, wrinkles, and ridges^17^,^18^,^19^. Biedermann *et al.* suggested that they were caused by compressive strain^20^ induced during the cooling process, due to the difference in the thermal expansion coefficient of epitaxial graphene and its supporting SiC substrate. Several studies were performed to understand the structural properties and the dynamic characteristics of these defects^21^,^22^,^23^. Subsequently, they were also observed on monolayer graphene grown on the Si-face^24^. Beside the morphology modifications of the graphene film, the curvature associated with these corrugations has been predicted to alter graphene's electronic and structural properties^25^,^26^. Although they are usually regarded as defects, and therefore as undesirable, it was reported that the wrinkles could be exploited for inducing pseudo-magnetic fields^27^, creating chemically reactive sites^28^, and for specific applications such as optical lenses^29^.

Here, we investigate the effect of such corrugations on epitaxial trilayer graphene grown on 6H-SiC(0001). Scanning tunneling microscopy and spectroscopy have allowed us to study the effect of wrinkles on the structural and electronic properties of epitaxial trilayer graphene on SiC. Distinct protrusions, identified as flower-shaped domains and isolated wrinkles were observed over the sample surface and were associated with the deformation of the standard hexagonal bonding within the carbon lattice. Moreover, the results of STM/STS mapping show that these flower-shaped domains have distinctly different electronic properties when compared with their adjoining flat regions. Furthermore, the wrinkle-like structures can be rotated by 60° during STM/STS measurements, confirming that these features are flexible.

## Results

### Growth of trilayer graphene and its characterization

**Figure 1:**
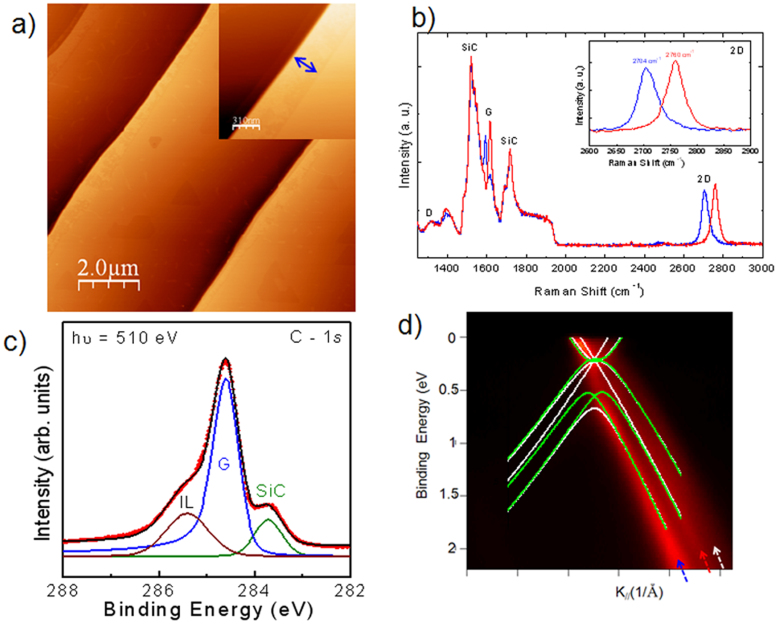
Structural and Electronic Properties of Epitaxial trilayer graphene. (a) Typical AFM image of trilayer graphene, (b) Raman spectra of the graphene sample (blue line) and of step edge (red line). Contributions at the G and 2D bands are observed, together with a very low signal at the defect band D, (c) C *1s* XPS spectra for epitaxial graphene annealed at 1550°C at hν = 510 eV. XPS measurements were performed at ϕ = 45° emergency angle with respect to the sample normal, (d) ARPES spectra along the ΓK direction of the graphene trilayer, together with theoretical calculation of ABA and ABC stacking (white and green, respectively). From the shift of the Dirac Energy we extract E_D_ = 0.2 meV, measured with respect to the Fermi energy.

The graphene used in this study was obtained by annealing 6H-SiC at 1550°C in 800 mbar argon for 10 min [see Methods]. The typical morphology of the graphene sample can be seen in the AFM image displayed in Figure 1(a). The surface was highly uniform over a large scale with atomically flat, 2–4 μm large terraces and 6–10 nm high steps. On defect-free areas of the sample, the terraces typically extended undisturbed over 50 μm in length. The step direction and terrace width were determined by the incidental misorientation of the substrate surface with respect to the crystallographic (0001) plane. As can be seen in the inset, at the step edges we observed the appearance of different regions which corresponded to multilayer ribbons located at the very edge of the terrace. These ribbon regions at the downward edges of the terraces corresponded to quadrilayers and in some cases five layer graphene (inset in Figure 1(a)). The average width of these ribbons was estimated to be about 500 nm, and their height usually less than 0.3 nm. This showed that the nucleation of new graphene layers started at the step edges of the substrate.

In addition to acquiring AFM images, we characterized the graphene layer using Raman spectroscopy^30^,^31^,^32^. In Figure 1(b), two typical spectra are shown, which correspond to measurements made on a terrace (blue curve) and on a step edge (red curve). Several intense peaks were observed between 1200 and 1800 cm^−1^, corresponding to second-order Raman bands originating in the SiC substrate. Graphene contributions were also observed, identified by three main structures: i) the D band at 1355 cm^−1^, ii) the G band (symmetric *E*_2*g*_ phonon mode) at 1595 cm^−1^ and iii) the 2D band at 2720 cm^−1^. In both spectra, the D band, which corresponds to disorder, was weak in comparison with the G and 2D bands (double resonant electron-phonon process). The low intensity of this peak showed that there was only a small number of defect/disorder in the graphene structure. This was an indication of the high quality of the epitaxial graphene produced. The G and 2D lines of the spectrum recorded on the step edge had blue shifted with respect to those of the spectrum obtained for the terrace. It is known that the position of both peaks depends on strain^33^ and carrier density. This shift shows a strong correlation between the strain and charge density in epitaxial graphene, with increases in compressive strain causing a decrease in electron doping. Thus, the observed blue shift of both lines indicated that there was a difference in strain (associated with the shift of the G and 2D lines) and carrier density (associated with the shift of the G line) between the two surface regions.

We probed the electronic properties of the sample using X-ray Photoelectron Spectroscopy (XPS) and Angle Resolved Photoelectron Spectroscopy (ARPES) experiments (Figure 1c and d). The C *1s* XPS spectrum of the sample, collected for hν = 510 eV, is shown in Figure 1(c). Due to the low electron inelastic mean free path at this photon energy, only the 2–5 first topmost layers were probed. A sharp C *1s* peak, labeled G, located at 284.5 eV in binding energy, indicates the presence of sp^2^ hybridized C–C bonds. The C *1s* spectrum showed three components at 283.4, 284.4, and 285.3 eV in binding energy^32^,^34^. These components corresponded to the SiC bulk (labeled SiC), the graphene layer (labeled G), and the interface layer (labeled IL), respectively. In order to estimate the thickness of the graphene film from XPS data, assuming the graphene-SiC sample could be modeled as a semi-infinite SiC substrate with a uniform graphene overlayer, the thickness was calculated from the ratio between the intensities of the G and SiC components. This ratio corresponded to an exponential decay of roughly 3.5 Ml of carbon covering. Moreover, no structure appeared at ~286.7 in C-1s XPS spectra, usually attributed to the presence of COOH groups, as a result of contamination and/or oxidation, meaning that even if the samples were prepared under high pressure conditions (P = 800 mbar), the graphene layers were very inert and did not show any contamination in this moderate vacuum. The band structure of the trilayer graphene was examined by ARPES measurements around the *K* point of the first Brillouin zone. Figure 1(d) shows the ARPES data revealing the electronic structure in the vicinity of the *K*-point of the Brillouin zone of graphene. The detailed dispersion of the different π-bands was well resolved, as shown in the 2D plots in panel (c). By counting the number of π-bands, three graphene layers could clearly be distinguished. In order to extract more information about the stacking geometry in our sample, we shifted the energy reference previously calculated (*ab initio*) band structures of both ABA and ABC stacked trilayer films^35^.The results are plotted on Figure 1(d) to give a comparison with the theoretical data. Our ARPES measured band structure was quite complex, making any obvious correspondence difficult to see. Even though such a comparison give any definitive information about the stacking, however we can safely conclude that our ARPES data represented an averaging between ABA and ABC patterns^36^. We also estimated that a natural shift between E_F_ and E_D_ was roughly in the range of 200 to 400 meV.

### STM/STS measurements: flower-shaped domains in trilayer graphene

**Figure 2:**
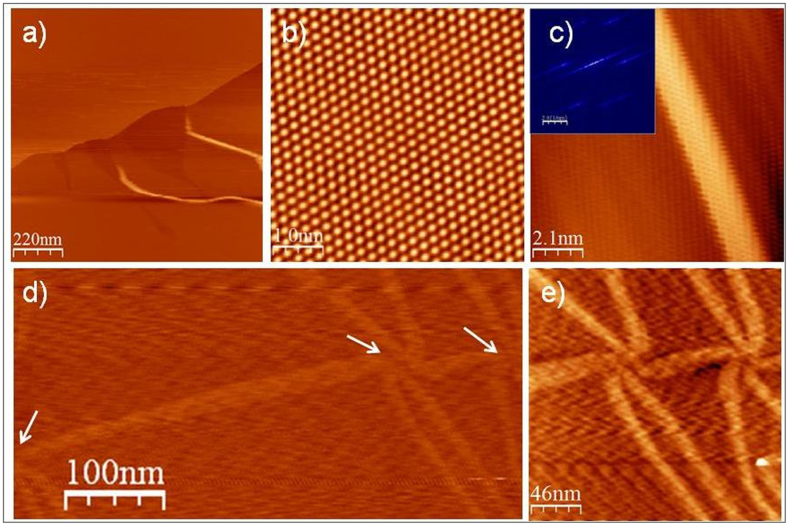
Morphology of trilayer graphene. (a) Large STM image (−1.7 V, 1 nA) of trilayer graphene (b) Triangular type structures (5 × 5 nm^2^), (c) An STM image of corrugated graphene with nearly periodic ripples of nanometer wavelength (inset Fourier transform of the STM image) (d) STM image of three interconnected flower-shaped structures, (e) STM image of two interconnected flower-shaped domains. V_s_ = 0.05 V, I_t_ = 200 pA) for (b - e).

To obtain detailed information about the morphology of the graphene trilayer, STM images of different surface regions were also acquired. Figure 2(a) shows typical STM images of the as-grown trilayer graphene. The graphene layers covered the large, atomically flat terraces that have surface areas of a few square micrometers. However, bright stripes, the so called wrinkles, appeared randomly along the step edges and across terraces and could also terminate on terraces, as well at the step edges. These wrinkle features were attributed to the 2D compressive stresses that developed upon cooling from the growth temperature. These features are consistent with the wrinkles reported in earlier studies of epitaxial graphene on C-face SiC^20^: Figure 2(b) is a high-resolution STM image of trilayer graphene in which the underlying (6 × 6) phase is completely hidden beneath the carbon lattice. The periodicity of this structure is equal to 2.5 Å, which is in good agreement with the (1 × 1) graphene lattice^14^,^32^. This graphene lattice appears triangular, indicating that only one of the two graphene atomic sublattices was imaged, due to the ABA Bernal stacking or ABC rhombohedral stacking between the three graphene layers.

When the trilayer graphene was imaged, various linear defects were found over the surface. We revealed two interesting types of protrusion: isolated wrinkles and flower-shaped domains. Figure 2(c) shows STM images of an isolated wrinkle structure on trilayer graphene. The graphene layer appears corrugated with nearly periodic ripples of nanometer wavelength. The average width of these ripples was estimated to be about 2 nm, and their height usually on the order of 200 pm. It is worth noting that the observed graphene wrinkles were often found to be accompanied by small corrugations (ripples) known to be intrinsic to graphene^17^. Fourier transform of the STM images (shown as insets) of Figure 2(c) showed that the surface exhibited an ordered structure with (1 × 1) symmetry of the graphene layer. From the FTs, we measured spatial periodicities of 2.5 Å for the surface. Figures 2(d) and (e) show a large STM image where we identified three interconnected flower-shaped domains. The average width of the middle of the flower was estimated to be about 15 nm, and their height usually less than 25 pm. The spiral form of the flower-shaped domains is attributed to the presence of a localized defect of the graphene layer which forms at the six-fold scattering center of the defects, such as networks of partial dislocations between ABA and ABC stacking in trilayer graphene.

**Figure 3:**
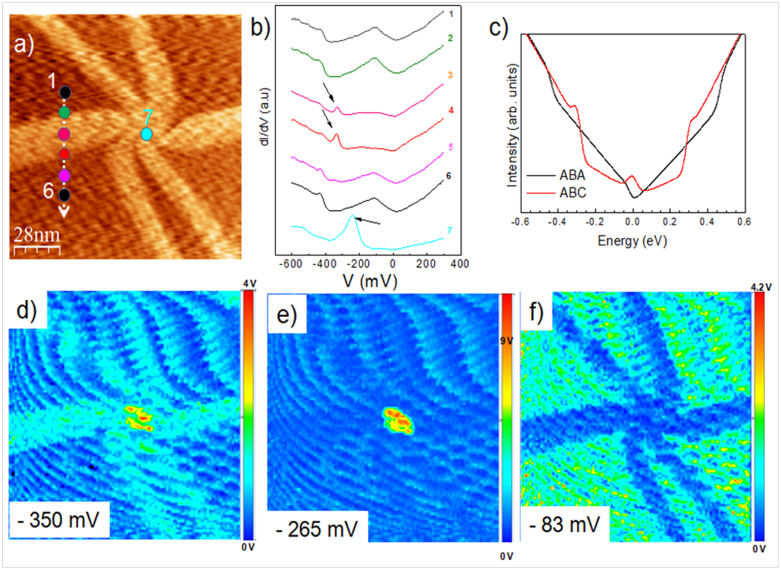
Flower-shaped domains on trilayer graphene. (a) STM topography showing the flower-shaped domains (*V*_s_ = −0.05 V, *I*_t_ = 0.2 nA). (b) Spatially averaged *dI*/*dV* curves in five different regions of the graphene indicated in (a). The curves show two -resonance peaks located at −344.4 mV and −264.5 on wrinkles and in the middle of the wrinkles. (c) The calculated total density of states of ABA and ABC stacking; the LDOS of ABC stacking has an additional resonance peak located at the Dirac level in comparison with the LDOS of ABA stacking. (e–f) *dI*/*dV* maps at sample voltages −344.4 mV, −264.5 and −83.4 mV, respectively, showing the transition between graphene and flower-shaped domains. For all the images, the current was stabilized at the parameters in (a) and the feedback was then switched off. The maps were recorded using lock-in detection.

The STM image in Figure 3(a) shows a single flower-shaped domain structured in a symmetric core of six connected domains. The average width of this shape of domain is estimated to be about 20 nm, and its height is also 25 pm^37^. The exact nature of this unique structure is currently under investigation, since the possible breaking of the perfect honeycomb lattice is expected to have profound effects on the electronic and mechanical properties of the grown graphene. For instance, defect engineering of graphene layers can produce localized one-dimensional metallic states within the carbon lattice for use in electronics.

In addition to their specific structural features, the flower-shaped graphene domains exhibited a singular electronic structure, as demonstrated by the STS measurements, shown in Figure 3(b). STS probed the electronic properties of the surface proportional to the local density of states (LDOS)^38^. Each spectrum was averaged over 20 STS spectra and taken at different cross locations on the flower-shape (spectra 3 and 4), flower middle (spectra 7) and on the flat graphene trilayer (spectra 1, 2, 5 and 6). The spectra obtained on the flat part were characteristic of graphene, with one minimum at the Fermi level (V_s_ = 0 mV) and another at the Dirac point (V_s_ = −360 mV). Interestingly, the STS spectrum obtained in the middle and on the petal-shaped domains of the flower differed from those obtained on the adjoining flat region. In addition to the expected STS of a trilayer graphene, there was a resonance peak located −264.5 mV and −344.4 mV below the Fermi level in the valence band, in the vicinity of our simple ARPES estimate of the position of the Dirac point energy. The first peak located at −264.5 mV, confirmed the presence of a strongly localized defect in the middle of the flower. Recent theoretical calculations have shown that such a peak can be induced by a highly localized impurity on graphene, causing a perturbation in the onsite potential energy at its location^39^. The position of the peak in the valence band suggested that this impurity had a repulsive potential (spectra 7 at −264.5 mV), as an attractive impurity potential would draw holes away from the regions of graphene in its vicinity.

This observation is compatible with a strongly localized defect at the partial dislocations between ABA and ABC areas of the graphene. The second peak localized at −344.4 mV (spectra 3 and 4) could be associated with an ABC structure embedded within an ABA trilayer graphene. When compared to the usual ABA structure, ABC-stacked few-layer graphene exhibits unusual band dispersions near the Dirac level. Specifically, the low-energy bands near the Dirac level (flat band) in ABC-stacked few-layer graphene has surface states with wave functions distributed on either *α* or *β* atoms of the outermost layers. To this end, theoretical LDOS derived from Density Functional Theory (DFT) calculations describing the ABA and ABC trilayers as shown in Figure 3 (c). The local density of states (LDOS) containing a resonant peaks located at the Dirac level for ABC structure in comparison with the LDOS of ABA stacking. Since in the experiment, the Fermi energy (E_F_) is shifted from the Dirac point (E_D_) due to substrate doping, the Dirac point in the calculated LDOS has not been aligned to the experimental position of E_D_ in our theory spectra. Therefore, only the relative positions at the Dirac point of the peaks are relevant, suggesting a good agreement between the theoretical LDOS (Figure 3(c)) and the experimental STS (Figure 3(b)). It is worthwhile noting that the size of this proposed ABC domain (20 nm) and the shape of the domain boundaries were also in agreement with recently reported results^40^,^41^. Moreover, equivalent signatures have been observed on AB-BA transitions in bilayer^42^, as also found here, since we think that an ABA-CBA transition occurs at the shape domain boundary. Beside these similarities, these flower-shaped domains could be stacked in an ABC structure.

We spatially mapped the LDOS at three resonance peaks located at −344.4 mV, −264.5 and −83.4 mV (Figure 3(b)). The graphene region showed uniformly higher conductance, in stark contrast to the flower-shaped region. It is interesting to note that there was an abrupt electronic change at the boundary between the ABC domain and the graphene layer (Figures 3 d and f). In addition, STS measurements performed at varying distances from the center of the protrusion indicated quite a sharp decay of this resonance peak at points beyond the edge of the ABC domain, indicating a highly localized potential perturbation. This observation is compatible with a strongly localized defect at the middle of the flower. It should be noted that no attention should be paid to the artifactual wavy background noise due to lock-in amplifier acquisition time parameters.

### STM/STS measurements: wrinkles in trilayer graphene

**Figure 4:**
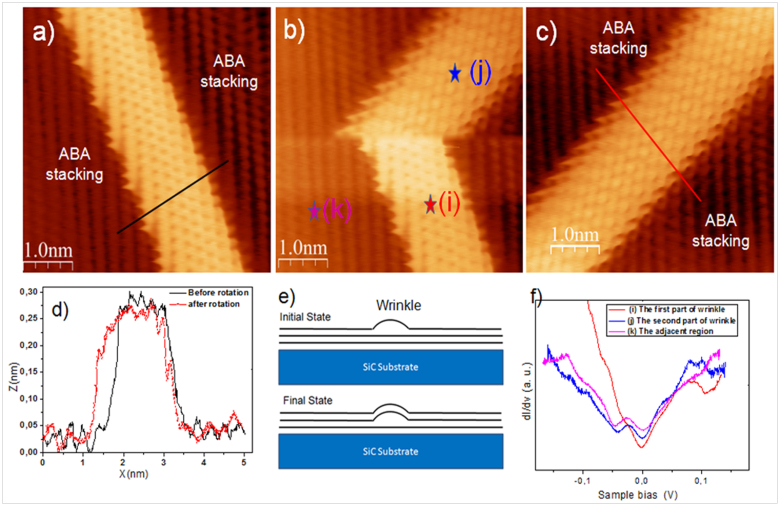
Different configurations of the wrinkles. (a) STM image taken on an isolated wrinkle (*V*_s_ = −0.05 V, *I*_t_ = 0.2 nA): honeycomb structure on the wrinkle and triangular pattern on the adjoining graphene region; (b) STM image (*V*_s_ = −0.05 V, *I*_t_ = 0.2 nA) showing the partial rotation of the wrinkle under a tip-surface interaction effect (c) STM image (*V*_s_ = −0.05 V, *I*_t_ = 0.2 nA) showing that the wrinkle has entirely rotated by 60° from its original orientation; (d) Height profiles corresponding to the black (before rotation of the wrinkles) and red (after rotation of the wrinkles) lines in (a and c); (e) Schematic showing the modification of the wrinkle after the rotation; (f) Spatially averaged *dI*/*dV* curves at three different regions in figure 4(b).

Recently, several theoretical studies have discussed either the rotation or the translation of graphene layers on graphite or graphene substrates^43^,^44^. To date, very few experimental results have been reported about the dynamics of free graphene layers and their motions when displaced out of their equilibrium configuration^45^. Figure 4 (a, b and c) shows three successive STM scans recorded using the same scanning tunneling parameters of an area with a time interval of 10 min. These images were acquired with a sample bias voltage V_s_ = 0.05 V and tunneling current I_t_ = 200 pA. As can be seen, the position of the overlying wrinkle changed substantially, being rotated out of the area by 60° after occasional spectroscopy measurement instability. Figure 4(a) indicates the atomic-resolution images of the wrinkle, here only a honeycomb lattice is observed, suggesting that the local curvature of the ripples did not break the 6-fold symmetry of the graphene lattice. However, over the entire region adjoining a wrinkle, the graphene appeared with a triangular lattice pattern. The distance separating two successive rows in the periodic arrangement was ~0.25 nm, indicating that the honeycomb 6-fold symmetry of the graphene lattice was broken and only three of the six carbon atoms in each hexagonal ring were observed.

The STM image in Figure 4(b) displays significant and sudden modifications of the wrinkle structure. The wrinkle had rotated by 60° with respect to its initial direction. Indeed, the lower part seemed to have kept the same orientation and identical structural characteristics (height and width) of those of the wrinkle presented in Figure 4(a). After rotation, the wrinkle became distinctly larger. The STM Image in Figure 4(c) shows that the wrinkle has fully turned, seeming to undergo a flattening of about 6% and an increase in its area of approximately 11%, as can be seen in the line scans presented in Figure 4(d). Surprisingly, in the upper part of wrinkle shown in Figure 4(b) and on the wrinkle in Figure 4(c), the honeycomb 6-fold symmetry of the graphene lattice seemed to be broken. The periodic arrangement defined by the sharper rows, displayed the same “three-for-six” patterns already observed in the regions adjoining the wrinkles. A possible explanation for this 3-fold symmetry could be the formation of a Bernal stacked bilayer on the graphene wrinkle. The force exerted by the STM tip during a spectroscopy measurement or voltage pulses can be strong enough to push the graphene so that the uppermost layer becomes coupled to the underlying graphene layer, forming a double layer wrinkle. This is indeed consistent with the appearance on our STM images of only three of the six carbon atoms in each hexagonal ring, as shown in the sketch in Figure 4(e). These observations show that during spectroscopy measurements or voltage pulses, wrinkles can be manipulated via STM-tip-surface interactions. To estimate the value of the van der Waals interaction between the tip and the graphene wrinkles, we considered the same geometry as ref^45^. The van der Waals force was estimated as F = AR/6D^2^, which corresponded to a 1 nN, where A is the Hamaker constant (1 × 10^−19^ J), R the STM tip radius (30 nm) and D the tip - sample distance (0.7 nm). In some cases, the force generated by such interactions, seems to be sufficient to make some corrugations disappear or create new ones.

Focusing now on the electronic properties of the wrinkles before and after the rotation, Figure 4(f) illustrates the STS data measured across a graphene/wrinkle boundary, as shown in Figure 4(b). The red curve shows a typical STS spectrum of the initial state of the wrinkles. The linear DOS around the Dirac point *E*_D_ is consistent with that of pristine graphene. The Dirac point *E*_D_ was in the same position as the Fermi level, suggesting very low charge transfer between the graphene and the substrate. This is consistent with the tunneling spectrum of quasi-free standing graphene monolayer. The curves (j, k) in Figure 4(f) which were measured in the region adjoining the wrinkle and the rotated wrinkle, respectively, shows distinct characteristics compared with those obtained on the initial wrinkle (curve (i) in Figure 4(f)). In addition to a soft minimum observed at zero bias, a pronounced dip is clearly resolved at V_D_ = −50 mV. The position of the Dirac point *E*_D_ is slightly above the Fermi level, suggesting a charge transfer between the graphene and the substrate. It is interesting to note that the shapes of the two spectra (j and k) are identical, showing a clear correlation between the structural and the local electronic properties of the corrugations observed. The STS measurements agree with the STM morphological observations reported in Figure 4 (a, b, c) and confirm the hypothesis described in the diagram shown in Figure 4(e), i.e. a force exerted by the STM tip may elevate the underlying graphene layer closer to the layer forming the wrinkle.

## Discussion

In the case of heteroepitaxial growth, the natural strain in the epilayers is a result of the misfit in lattice parameters and thermal expansion coefficient and of the plastic relaxation process. Epitaxial trilayer graphene on 6H-SiC(0001) can be regarded as a model system to study the interplay between epitaxy and changes in the resulting crystal structure. Trilayer epitaxial graphene on SiC is an extreme example of heteroepitaxial systems because this combined material has a large lattice misfit and a difference in crystal symmetry (Bernal and rhombohedral stacking). This residual strain can induce a myriad of defects on the atomic-scale, on grain boundaries and on miscellaneous forms of corrugation; ripples, wrinkles, ridges and partial dislocations are encountered in all epitaxial multilayer graphenes. Partial dislocations in trilayer graphene cause direct changes in the crystal structure, in our case, for example, from Bernal stacking to rhombohedral stacking (ABA-ABC). Concerning the origin of the dislocations, our STM studies show very small holes and the spiral structure of ABC stacking indicate that the origins of the dislocations must already be present at the graphene/SiC interface. Most likely, the dislocations and the wrinkles form as a result of residual strain either during growth of the graphene on the SiC or during cooling of the samples from 1550°C to room temperature.

Concerning the structural properties of wrinkles, our results suggest that these structures are flexible and the interaction with the STM tip causes an occasional rotational instability of the wrinkles during the spectroscopy measurement. Several STM experiments were performed to understand the dynamics of such features. From all the STM images taken sequentially, we noticed that a wrinkle can rotate under STM-tip effects, but its orientation is always found to be close to a high symmetry direction in the graphene lattice^44^. Recently it has been shown that a wrinkle can separate the gradual and continuous transition from Bernal (ABA) to rhombohedral (ABC) stacking^46^, where the ABA and ABC domains are constructed from a continuous graphene layer. In this configuration, it is necessary to have a wrinkle separating the two domains to accommodate the shift between the two stacking orders.

Careful examination of the STM images (Figure 4(a)) reveals that the hexagonal arrangement of the carbon layer and its orientation remains undisturbed around the wrinkle boundary, as confirmed by the nearly identical Fourier transform images. Triangular lattice symmetry consistent with multilayer graphene was observed in these two regions of the STM image. In addition, the STS spectra show a similar behavior of the flat regions adjoining the wrinkle. The typical V shape observed in the STS spectra (Figure 4(f)) indicates that this is a Bernal stacked multilayer graphene. As shown in Figure 4(c), to the left and the right of the wrinkle, the atomic arrangements do not show a change before and after the wrinkle rotation. This confirms that the surface presents a single stacking (ABA) over the whole image.

**Figure 5:**
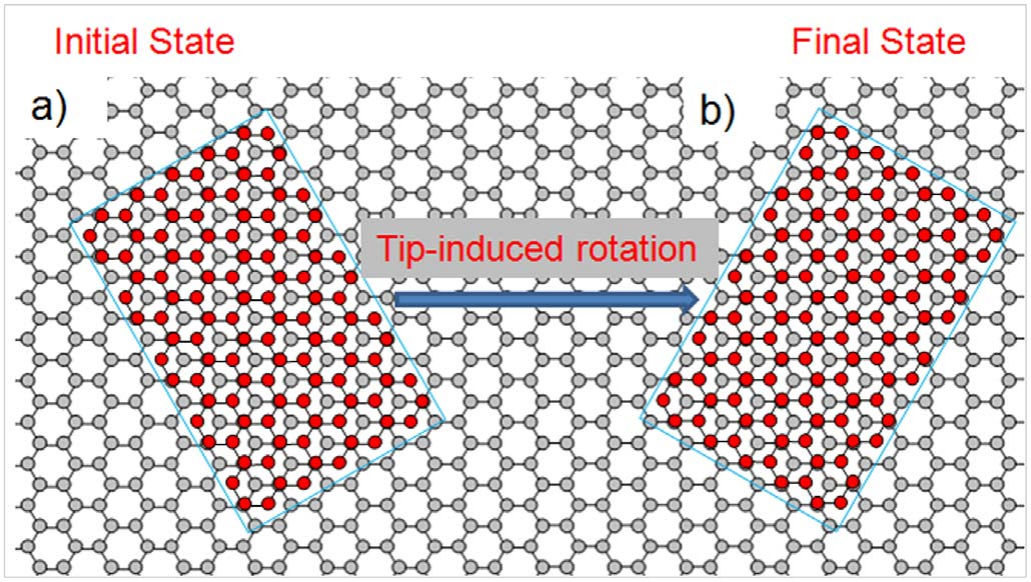
Structure of the wrinkles on the graphene layer. Schematic representation of the proposed diffusion mechanism through rotation of the wrinkle from the initial to the final states: (a) initial state (b) final state of the wrinkle.

Therefore, we propose that the wrinkle could switch by rapid rotation from an initial state to a stable state in registry with the underlying graphene layer, as schematically illustrated in figure 5. A schematic model of edges of armchair graphene is in good agreement with our measured STM image of the wrinkle (Figure 4(a)). The armchair edges are also shown by standing wave patterns of bright rings in the proximity of the edges (Figure 4(c). We note that the relative stability of zigzag versus armchair edges in graphene has been theoretically debated with different conclusions reached after different calculations. Experimentally, both armchair and zigzag oriented edges have been found in graphene prepared by different methods. Epitaxial graphene wrinkles appear to preferentially show armchair edges. On the other hand, graphene layers grown on metal surfaces tend to show zigzag edges^47^, suggesting that the stability of zigzag versus armchair edges may depend on various factors, such as the local environment of the graphene. We expect our findings to contribute to the understanding of wrinkle stacking and its role in tailoring the structural and electronic properties of trilayer graphene.

In summary, we have combined complementary techniques to investigate the structural and electronic proprieties of nanostructures present in epitaxial trilayer graphene. These nanostructures are interconnected flower-shaped domains and isolated wrinkles that are in fact bulging regions of the graphene layer, which appear to relieve the compressive strain induced by a mismatch in the thermal expansion coefficients of the graphene layer and its supporting SiC substrate. The flower-shaped domain is attributed a partial dislocations between ABA and ABC stacking in trilayer graphene. Our STM/STS study revealed that the wrinkles are preferentially oriented close to the high symmetry direction in the graphene lattice and they have distinctly different structural and electronic proprieties in comparison to the adjoining flat regions of trilayer graphene. Furthermore, we have found that it is possible to manipulate these corrugations and even create new ones through the tip-surface interactions during scanning, voltage pulses or spectroscopic instabilities.

## Methods

### Growth of graphene

The graphene trilayers studied in this paper was produced via a two-step process beginning with a starting substrate of 6H-SiC(0001). Prior to graphitization, the substrate was hydrogen etched (100% H_2_) at 1550°C to produce well-ordered atomic terraces of SiC. Subsequently, the SiC sample was heated to 1000°C in a semi UHV chamber and then further heated to 1550°C in an Ar atmosphere. This graphitization process resulted in the growth of an electrically active graphene layer on top of the buffer layer, covalently bound to the substrate. The sample is cooled down to room temperature and transferred *ex-situ* to perform different measurements. Before STM/STS and XPS/ARPES, the sample is outgassed at 600°C for one hour at a pressure of 5 × 10^−10^ mbar.

### Characterization

The graphene sample obtained was characterized using various sensitive techniques: Raman and angle resolved photoemission spectroscopy, atomic force microscopy (AFM), and scanning tunneling microscopy and spectroscopy (STM/STS). The micro-Raman spectroscopy was performed at room temperature with a Renishaw spectrometer using 532 nm excitation wavelength laser light focused on the sample using a DMLM Leica microscope with a 50× objective and a power of 5 mW with spot size of about 1 μm. The AFM measurements were carried out under ambient conditions and the images were recorded in non-contact mode.

The XPS/ARPES measurements were performed on the Tempo beamline at the Soleil synchrotron (Saint-Aubin, France). The photon energy (hv = 50 eV) and sample orientation were set in order to explore the k space region around the K point of the first Brillouin zone.

STM/STS measurements were carried out using an Omicron ultrahigh vacuum low temperature scanning tunneling microscope (UHV-LT-STM). STM/STS measurements were acquired at 4.2 K in the constant current mode for different bias voltages V applied to the sample. For the STS measurements, performed at *T* = 4.2 K, the I(V) characteristics were acquired while the feedback loop was inactive, the differential conductivity dI/dV (V, x, y), proportional to the LDOS, was measured directly by using a lock-in technique. For this purpose a small AC modulation voltage *V*_mod_ was added to *V* (*V*_mod,p-p_ = 10 mV, *f*_mod_ = 973 Hz) and the signal *dI/dV* detected using a lock-in amplifier in order to determine the differential conductivity *dI*/*dV*_mod_. Our STM tips were prepared from 0.25 mm W polycrystalline wire. They were electrochemically etched in a NaOH solution and then passivated in an HF bath. The quality of this etching procedure and the global apex shape was checked using scanning electron microscopy. The tips were then introduced into an ultrahigh vacuum preparation chamber connected to the STM chamber. There, they were flashed by Joule heating to temperatures up to white light emission. This procedure allowed the removal of contaminants and oxides from the apex.

## Author Contributions

B.L. and A.O. grow the graphene sample, E.P. characterized it by AFM and Raman spectroscopy. A.O., M.G.S. and F.S. conducted the measurements by XPS and ARPES. J.C.G. and C.D. carried out STM/STS experiments. B.L., E.P., S.L., J.C.G. and A.O. analyzed the data. B.L. and A.O. wrote the paper with all authors contributing to the final version. A.O. planned the experiments and supervised the project.
